# Appropriateness of antibiotic management of uncomplicated skin and soft tissue infections in hospitalized adult patients

**DOI:** 10.1186/s12879-016-2067-0

**Published:** 2016-11-29

**Authors:** Thomas L. Walsh, Lynn Chan, Chelsea I. Konopka, Michael J. Burkitt, Matthew A. Moffa, Derek N. Bremmer, Monika A. Murillo, Courtney Watson, Noreen H. Chan-Tompkins

**Affiliations:** 1 Department of Medicine and Division of Infectious Diseases, Allegheny General Hospital, 320 East North Ave. East Wing Office Building, Suite 406, Pittsburgh, PA 15212 USA; 2 Department of Medicine and Division of Infectious Diseases, Western Pennsylvania Hospital, 4800 Friendship Avenue, Pittsburgh, PA 15224 USA; 3 Department of Pharmacy, Allegheny General Hospital, 320 East North Avenue, Pittsburgh, PA 15212 USA; 4 Division of Hospital Medicine, Allegheny General Hospital, 320 East North Avenue, Pittsburgh, PA 15212 USA; 5 Division of Hospital Medicine, Western Pennsylvania Hospital, 4800 Friendship Avenue, Pittsburgh, PA 15224 USA; 6 Department of Pharmacy, Western Pennsylvania Hospital, 4800 Friendship Avenue, Pittsburgh, PA 15224 USA; 7 Center for Inclusion Health, Allegheny General Hospital, 320 East North Avenue, Pittsburgh, PA 15212 USA

**Keywords:** Cellulitis, Skin and soft tissue infection, Subcutaneous abscess, Antibiotic utilization

## Abstract

**Background:**

Skin and soft tissue infections (SSTIs) are a leading cause for hospitalizations in the United States. Few studies have addressed the appropriateness of antibiotic therapy in the management of SSTIs without complicating factors. We aimed to determine the appropriateness of antibiotic treatment duration for hospitalized adult patients with uncomplicated SSTIs.

**Methods:**

This was a retrospective analysis performed at two academic medical centers in Pittsburgh, Pennsylvania on patients aged 18 years and older with primary ICD-9 code for SSTIs admitted August 1st, 2014–March 31st, 2015. The primary outcome was the appropriateness of antibiotic treatment duration for uncomplicated SSTIs. Secondary objectives included the appropriateness of antibiotic agent spectrum, duration of inpatient length of stay (LOS), utilization of blood cultures and advanced imaging modalities, and re-hospitalization for SSTI within 30 days of discharge from the index admission.

**Results:**

A total of 163 episodes were included in the cohort. The mean duration of total antibiotic therapy was 12.6 days. Appropriate duration was defined as receipt of total antibiotic duration of less than 10 days and occurred in 20.2% of patients. Twenty eight percent of patients received antibiotics for greater than 14 days. Seventy three (44.8%) patients received greater than 24 h of inappropriate extended spectrum gram-negative coverage; 65 (39.9%) received anaerobic coverage.

**Conclusions:**

In the majority of patients, treatment duration was excessive. Inappropriate broad spectrum antibiotic selection was utilized with regularity for SSTIs without complicating factors. The management of uncomplicated SSTIs represents a significant opportunity for antimicrobial stewardship.

## Background

Skin and soft tissue infections (SSTIs) are the second most common type of infections leading to hospitalization in the United States and are becoming increasingly more prevalent [1, 2]. Hospitals have experienced a 71% increase in the rate of hospitalizations due to SSTIs recently, and these infections are associated with significant healthcare costs [2–5]. Given the substantial impact these infections play in healthcare consumption, it is imperative that evidence-based strategies be developed and implemented to optimize patient outcomes and utilization of healthcare resources while limiting the unintended consequences of unnecessary antibiotic use. Given the rising plague of antimicrobial resistance, strategies to limit the utilization of our limited armamentarium of antimicrobial agents are greatly needed. Given their breadth of effect and significant impact on morbidity and mortality, multidrug resistant bacteria are considered one of the largest threats to public health and national security by numerous prominent organizations including the Institute of Medicine, the Center for Disease Control and Prevention Task Force on Antimicrobial Resistance, and the Infectious Disease Society of America (IDSA) [6–9]. In its 2013 annual report on global risks, the World Economic Forum concluded that “arguably the greatest risk…to human health comes in the form of antibiotic-resistant bacteria” [10].

Current evidence has demonstrated that 5–7 days of antimicrobial therapy is adequate for clinical cure of uncomplicated SSTIs [11–14]. Despite this, treatment duration in real life settings is inappropriately long, with prolonged courses of up to 2 weeks or more commonly prescribed [15–20]. The predominant pathogens causing uncomplicated SSTIs are aerobic gram-positive organisms, namely *Staphylococcus aureus* (*S. aureus*) and β-hemolytic streptococcal species [15, 21–28]. Despite this, patients are frequently treated with broad spectrum antibiotic therapy with activity against aerobic gram-negative organisms and anaerobic bacteria [15, 16, 18–20]. The appropriate use of antimicrobials is critical as unnecessary utilization is intricately associated with patient harm due to increased drug resistance, adverse drug events/toxicity, and collateral damage including *Clostridium difficile* (*C. difficile*) infection [6–10, 29–32]. Despite the burden of SSTIs on their contribution to overall antibiotic use in hospitalized patients, knowledge of current antibiotic prescribing practices is limited. Further studies evaluating the details of antibiotic agent spectrum and duration of therapy for SSTIs without complicating factors are needed in order to properly develop targeted initiatives in order to optimize antibiotic utilization for this disease state.

The purpose of this retrospective cohort study was to assess the appropriateness of antibiotic duration and spectrum for SSTIs in patients without complicating factors and identify opportunities for antimicrobial stewardship through a thorough descriptive analysis of the evaluation, treatment, and outcomes among a cohort of patients hospitalized for uncomplicated SSTIs at two large teaching facilities.

## Methods

### Study setting and population

Allegheny General Hospital (AGH) is a 631 bed quaternary care teaching facility with approximately 22,000 inpatient admissions yearly. The Western Pennsylvania Hospital (WPH) is a 317 bed community based teaching hospital with nearly 6,800 inpatient admissions annually. Both facilities are located in Pittsburgh, Pennsylvania and are members of the Allegheny Health Network (AHN). The evaluation was approved and granted exempt status from the AHN Institutional Review Board as it was deemed Quality Assessment/Quality Improvement.

### Study design

We identified patients discharged from AGH and WPH from August 1st, 2014 through March 31st, 2015 with a primary diagnosis of SSTI using International Classification of Diseases, Ninth Revision (ICD-9), coding data. The search codes included cellulitis and cutaneous abscess (681), other cellulitis and abscess (682), acute lymphadenitis (683), other infections of skin and subcutaneous tissue (686), and erysipelas (035). All patients with one of these primary ICD-9 codes were identified and electronically extracted via our Quality Intelligence department. For patients with multiple hospitalizations at AGH and WPH for SSTI during the study period, each episode was reviewed and included for analysis. Encounters limited to the Emergency Department were not included. Demographic information, admission and discharge dates, and length of inpatient hospitalization were extracted electronically via our Quality Intelligence department. Utilizing a standardized data collection instrument, study investigators (TLW, LC, CIK, MJB, MAM, DNB, MAM, NHCT) verified the discharge diagnosis and obtained information regarding patient comorbidities, microbiologic data, radiographic studies, inpatient and outpatient antimicrobial therapy, and subsequent inpatient clinical encounters at AGH and WPH during the 30 days following hospital discharge via review of the patients’ electronic medical record and daily progress notes written in the patients’ paper medical record. Inpatient antibiotic therapy was determined via review of the electronic medication administration record. Outpatient antibiotic therapy was determined via review of the electronic medication reconciliation form in each patient’s transition of care document.

Patients were excluded for age less than 18 years, transfer from an outside hospital, left against medical advice (AMA), death during index hospitalization, or presence of a concomitant bacterial infection that required antibiotic therapy. For the purposes of the study aim, the term “uncomplicated” was used to describe cases of SSTI where there were no known traditional risk factors for infection due to gram-negative rods or anaerobic bacteria. Patients were excluded if they were associated with any of the following: management in an intensive care unit (ICU), peri-rectal involvement, peri-orbital involvement, associated with human or animal bite, odontogenic source of infection, associated with diabetic ulceration or chronic underlying ulceration, surgical wound infection, traumatic aquatic injury, associated with intravenous (IV) illicit drug use, concern for necrotizing infection, associated with osteomyelitis, presence of retained infected foreign body, presence of bacteremia, and presence of neutropenia or severe cell-mediated immunodeficiency. Severe immunodeficiency was defined as use of chronic immunosuppressive therapy at the time of admission (equivalent of > 10mg prednisone daily), human immunodeficiency virus (HIV) with cluster of differentiation 4 (CD4) cell count less than 350 per cubic millimeter, active malignancy with receipt of systemic chemotherapy within the 30 days prior to index admission, and receipt of prior solid organ transplant or hematopoietic stem cell transplantation. This definition of uncomplicated SSTI is similar to that used in prior studies [15, 17–20].

### Study outcomes definitions

All endpoints were specified prior to the evaluation. The primary outcome of interest was to determine the appropriateness of antibiotic treatment duration for SSTIs in hospitalized patients without complicating factors. Treatment duration of ten days or longer was defined as inappropriate. This definition is consistent with that used in prior real world evaluations [15, 18–20]. Duration of therapy was defined as the cumulative number of calendar days during which an antibiotic was administered in the inpatient setting or an antibiotic was prescribed to be administered as an outpatient following discharge. When calculating calendar days of therapy, the assumption was made that patients would initiate the outpatient antibiotic regimen on the day of discharge.

Secondary objectives included the appropriateness of antibiotic agent spectrum of coverage, the duration of inpatient length of stay (LOS), utilization of blood cultures and advanced imaging techniques via computed tomography (CT) scan or magnetic resonance imaging (MRI), and re-hospitalization for SSTI to AGH or WPH within 30 days of discharge from the index admission. Utilization of greater than 24 h of antibiotics with extended gram-negative activity, extended anaerobic activity, and anti-pseudomonal activity was defined as inappropriate unless the patient had a gram negative rod or anaerobic organism isolated.

Organisms (excluding coagulase-negative staphylococci, diphtheroids, *Micrococcus*, and *Proprionibacterium acnes*) were considered to be the etiology of purulent SSTIs when cultured from purulent drainage or an abscess cavity. As needle aspirates and punch biopsies are not routinely performed for cellulitis at AGH or WPH, cases of non-purulent SSTIs were not included in the evaluation of microbiologic etiology.

Antibiotics with extended gram-negative activity were defined as aztreonam; colistin; tigecycline; 3rd, 4th and 5th generation cephalosporins; β-lactam/β-lactamase inhibitor combinations (amoxicillin/clavulanate, ampicillin/sulbactam, piperacillin/tazobactam); carbapenems (ertapenem, meropenem); and fluoroquinolones (ciprofloxacin, levofloxacin, moxifloxacin).

Antibiotics with extended anaerobic activity were defined as β-lactam/β-lactamase inhibitor combinations, carbapenems, metronidazole, and tigecycline.

Antibiotics with anti-pseudomonal activity were defined as aztreonam, cefepime, ceftazidime, ciprofloxacin, colistin, levofloxacin, meropenem, and piperacillin/tazobactam.

### Data analysis

Differences between the AGH and WPH cohorts in the continuous variables of age and length of stay were assessed using the two sample t-test. *P* < 0.05 was considered significant. Differences between the AGH and WPH cohorts in the categorical variables of race, site of infection, presence of purulence, treatment duration, antibiotic agent spectrum, and utilization of blood cultures and advanced imaging modalities were assessed using Fisher exact. *P* < 0.05 was considered significant. Stata statistical software, version 12, was used for data analysis.

## Results

Two hundred eighty four patients with a principal discharge diagnosis of acute bacterial skin and skin structure infection during the project period were initially identified by ICD-9 codes. After manual review of the electronic health record, 121 (42.6%) patients were excluded from the analysis secondary to transfer from an outside hospital (38 [13.4%]), left against medical advice (16 [5.6%]), odontogenic infection (15 [5.3%]), human or animal bite (12 [4.2%]), intravenous drug use (17 [6%]), osteomyelitis (8 [2.8%]), surgical wound (8 [2.8%]), necrotizing infection (7 [2.5%]), diabetic and chronic ulceration (7 [2.5%]), immunocompromised host (5 [1.8%]), peri-orbital involvement (4 [1.4%]), peri-rectal involvement (4 [1.4%]), management in an ICU (4 [1.4%]), presence of a retained foreign body (4 [1.4%]), and active malignancy (2 [0.7%]). Some of the patients were excluded for more than one characteristic described above. No patients were excluded due to death or presence of bacteremia.

**Fig. 1:**
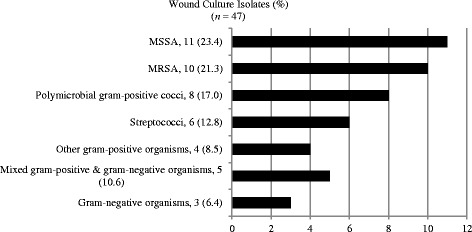
Wound culture isolates. *SSTIs* skin and soft tissue infections, *MSSA* methicillin-susceptible *Staphylococcus aureus*, *MRSA* methicillin-resistant *Staphylococcus aureus*

The final cohort included 163 (57.4%) unique episodes. The mean age was 55.3 (SD 19.2) years with the majority of patients being male 84 (51.5%) and Caucasian 132 (80.9%) (Table 1). The most common site of infection was the leg in 110 (67.5%) patients. Purulence was noted in 49 (30.1%) patients. A bacterial organism was isolated in 47 (28.8%) of patients. The main organism on culture was *S. aureus* with methicillin-susceptible *S. aureus* (MSSA) isolated in 11 (23.4%) and methicillin-resistant *S. aureus* (MRSA) in 10 (21.3%) (Fig. 1).

**Table 1:** Demographic and disease characteristics

Characteristic	AGH (*n* = 120)	WPH (*n* = 43)	Total cohort (*n* = 163)	*p* value
Mean Age (SD), *years*	55.6 (19.2)	54.6 (19.3)	55.3 (19.2)	.77
Male, *n* (%)	66 (55.0)	18 (41.9)	84 (51.5)	.16
Race, *n* (%)				.87
Caucasian	97 (80.8)	35 (81.4)	132 (80.9)	
African American	19 (15.8)	6 (14.0)	25 (15.3)	
Other	4 (3.3)	2 (4.7)	6 (3.7)	
Site of infection, *n* (%)				.07
Leg	85 (70.8)	25 (58.1)	110 (67.5)	
Arm	27 (22.5)	9 (20.9)	36 (22.1)	
Trunk	3 (2.5)	3 (7.0)	6 (3.7)	
Face	3 (2.5)	2 (4.7)	5 (3.1)	
Other	2 (1.7)	4 (9.4)	6 (3.7)	
Purulence, *n* (%)	37 (30.8)	12 (27.9)	49 (30.1)	.85
Mean length of stay (SD), *days*	3.8 (2.5)	3.3 (2.7)	3.7 (2.6)	.25

*AGH* Allegheny General Hospital, *SD* = standard deviation, *WPH* Western Pennsylvania Hospital

*p* value represents comparison between study sites

**Fig. 2:**
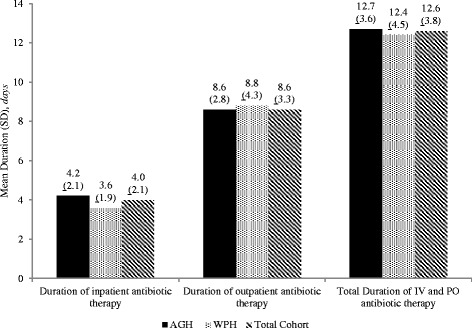
Antibiotic treatment duration. *AGH* = Allegheny General Hospital, *IV* intravenous route of administration, *PO* oral route of administration, *SD* Standard Deviation, *WPH* Western Pennsylvania Hospital

Appropriate duration of less than 10 days of total antibiotic therapy occurred in only 20.2% (33/163) of patients. Patients received 10-14 days of total antibiotic therapy 51.5% (84/163) of the time, and 28.2% received antibiotics for greater than 14 days (Table 2). The mean duration of therapy was 12.6 days and this was very similar between facilities (Fig. 2). The mean hospital length of stay was 3.7 days.

**Table 2:** Appropriateness of antibiotic treatment duration and selection

Variable	AGH (*n* = 120)	WPH (*n* = 43)	Total Cohort (*n* = 163)	*p* value
Appropriate treatment duration, *n* (%)				.74
Less than 10 days	22 (18.3)	11 (25.6)	33 (20.2)	
Inappropriate treatment duration, *n* (%)				.99
10 to 14 days	63 (52.5)	21 (41.9)	84 (51.5)	
More than 14 days	35 (29.2)	11 (25.6)	46 (28.2)	
Inappropriate broad spectrum antibiotics for > 24 h, *n* (%)				
Gram-negative coverage	52 (43.3)	21 (48.8)	73 (44.8)	.59
Anaerobic coverage	46 (38.3)	19 (44.2)	65 (39.9)	.59
Anti-pseudomonal coverage	21 (17.5)	7 (16.3)	28 (17.2)	.99

*AGH* Allegheny General Hospital, *WPH* Western Pennsylvania Hospital

*p* value represents comparison between study sites

Seventy three (44.8%) patients received greater than 24 h of extended spectrum gram-negative coverage; 65 (39.9%) received greater than 24 h of anaerobic coverage; and 28 (17.2%) received greater than 24 h of unnecessary anti-pseudomonal coverage. Duration of therapy and inappropriate use of broad spectrum therapy were similar between the two facilities (Table 2).

The 30-day re-admission rate for the entire cohort was 7.4% (12/163) with 8.3% (10/120) and 4.7% (2/43) of patients being re-admitted to AGH and WPH, respectively. Re-admissions secondary to recurrent SSTI occurred in 3.5% (6/163) of patients with all six re-admissions occurring at AGH. Of the 12 patients who were re-admitted within 30 days, 2 received less than 10 days of total antibiotic therapy originally, while 10 of the 12 patients received 10 days or more of total antibiotic therapy. The rates of 30 day re-admissions were similar between the group of patients who received less than 10 days of therapy (6.0%) and those who received 10 days or more of therapy (7.7%).

Of the total cohort of 163 patient episodes, 127 (77.9%) had blood cultures collected. Twenty nine patients (17.8%) in the total cohort received a CT scan, while 13 (8.0%) received an MRI. In total, 25.8% of patients received one of these advanced imaging modalities.

## Discussion

Treatment duration of 10 days or more was exceeded in the majority of patients, and we were quite surprised to learn that only 20.2% of patients received an appropriate duration of less than 10 days of total antibiotic therapy, while nearly a third of patients received greater than two weeks of therapy. While our findings are consistent with those of previous studies demonstrating that the median duration of antimicrobial therapy for hospitalized patients with cellulitis and cutaneous abscess was nearly two weeks [15–20], this remains startling given that the IDSA guidelines recommend 5 days of therapy with the caveat of extending the duration of therapy for patients who fail to improve after 5 days [12]. This recommendation is supported by a randomized trial of the treatment of cellulitis which demonstrated that 5 days of therapy was as effective as 10 days, although a paucity of patients required inpatient management [11]. While there is sparse data otherwise regarding the duration of therapy for hospitalized patients with uncomplicated SSTIs, the available evidence suggests these patients can be safely treated with short courses of antibiotics [13, 14, 17, 33]. Schuler and colleagues aimed to decrease the duration of antibiotics prescribed in children hospitalized for uncomplicated SSTIs by utilizing quality improvement methods. They accomplished this aim by increasing prescriptions for short courses of therapy, defined as 7 days or less, at discharge. There were 641 admissions for uncomplicated SSTIs over a 23 month period. The proportion of children discharged with 7 days or less of antibiotics increased from a baseline median of 23%–74%. Differences in the proportion of children who experienced treatment failure or recurrence before and after project initiation were not significant [17]. Jenkins and co-investigators implemented an institutional guideline to standardize and streamline the evaluation and treatment of inpatient SSTIs in order to optimize antibiotic utilization. This intervention led to a significant reduction in median duration of antibiotic therapy from 13 days to 10 days without an increase in clinical failure [33].

The diagnosis of SSTIs is based upon morphologic features of the lesions and the clinical setting. Cultures of needle aspirates are not indicated in routine care [12]. However, data from five series in the 1980s using needle aspiration elucidated common pathogens, with *S. aureus* and β-hemolytic streptococci accounting for the vast majority of infections [21–25]. Two studies in the late 1980s found the yield of punch biopsies to be superior to needle aspirates [23, 25]. In cases where a microbiologic etiology was identified, gram-positive cocci were present in all but one case [23, 25]. More recent studies continue to demonstrate that β-hemolytic streptococci and *S. aureus* continue to be the primary causes of uncomplicated cellulitis and cutaneous abscesses, comprising up to 97% of positive culture results [15, 28]. Additionally, the IDSA practice guidelines for the diagnosis and management of skin and soft tissue infections recommend the use of narrow spectrum antibiotics targeting only gram-positive pathogens for cases of uncomplicated SSTIs [12]. In our cohort, the term “uncomplicated” was used to define patients with SSTI who did not have traditional risk factors for infection due to gram-negative rods or anaerobic bacteria. Despite this, in our current evaluation where we excluded patients with known risk factors for SSTIs due to gram-negative and anaerobic organisms, we found that nearly half of our patients with uncomplicated SSTIs still received inappropriate therapy with extended gram-negative coverage and a similar number received extended anaerobic coverage. Our findings are similar to those reported elsewhere in the literature [15, 16].

Cultures of blood are unnecessary in typical cases of cellulitis, according to the IDSA guidelines [12]. Less than 5% of patients with cellulitis have positive blood cultures [21, 34, 35]. A retrospective analysis of blood cultures in over 500 patients with community-acquired cellulitis by Perl and co-investigators [35] found a relevant isolate in only 2% of blood cultures obtained, indicating that blood cultures were not likely to be cost effective for most patients with cellulitis. Per the IDSA guidelines, “blood cultures should be obtained…for patients with malignancy, severe systemic features (such as high fever and hypotension), and unusual predisposing factors, such as immersion injury, animal bites, neutropenia, and severe cell-mediated immunodeficiency” [12, 21]. In our current analysis, we aimed to include only patients without risk factors for gram negative or anaerobic infections. Therefore, we excluded patients with malignancy on chemotherapy, neutropenia, severe cell-mediated immunodeficiency, immersion injuries, and animal bites. We also aimed to include only patients without severe infection, so we excluded patients treated in an intensive care unit, concern for necrotizing infection and with deeper space infection. In our cohort of included patients, none had fever of greater than 39° Celsius and none had hypotension requiring vasopressor support. Therefore, no patients in our evaluation met criteria to have blood cultures obtained; despite this, blood cultures were collected in greater than three-quarters of our patients. Furthermore, none of the 121 patients who met exclusion criteria for our study had positive blood cultures, either. Additionally, radiographic examination is unnecessary in most cases of uncomplicated SSTIs [34]. Plain film radiographs and CT offer limited value except when the clinical setting suggests a subjacent osteomyelitis. Notwithstanding this, over a quarter of patients in our cohort received advanced imaging with CT or MRI. These are not cost-effective practices for patients with SSTI and represent potential opportunities to decrease resource utilization.

This evaluation has several important limitations. First, it was a retrospective analysis where case finding was reliant on ICD-9 coding from hospital discharge data, and this strategy may have led to underestimation of the true number of hospitalized patients with uncomplicated SSTIs at our institutions. Additionally, post-discharge data analysis was limited to re-admissions to AGH and WPH. Visits to other inpatient facilities, urgent care centers, and physicians’ outpatient offices may have been missed, leading to an inability to determine rates of treatment failure or the need to extend or re-introduce antibiotic therapy. We were also unable to assess compliance with outpatient antibiotics. Additionally, our inclusion criteria were intentionally selected to only include those hospitalized patients with SSTIs without complicating factors. Therefore, we cannot comment upon those patients with SSTIs with complicating factors. Lastly, duration of therapy was calculated via calendar days of administration. This may potentially lead to longer calculated total duration compared to a method utilizing defined daily doses or hours of therapy administered.

Our evaluation has numerous strengths as well. Our study focused upon two different centers with different patient populations and different medical care providers. The results were very similar between the facilities and are in line with the findings in other studies [15, 16]. Our findings demonstrate that inappropriate use of antibiotics in the management of SSTIs, both in duration and spectrum, was pervasive in a community hospital as well as in a large academic quaternary care center. This suggests that an opportunity for improved SSTI management exists at both type of facilities. Additionally, given our extensive exclusion criteria, we were able to analyze a homogenous patient population focusing only upon hospitalized patients with SSTI without traditional risk factors for infection with gram negative rods or anaerobic bacteria.

There is a paucity of literature examining the appropriateness of antibiotic spectrum and duration for hospitalized adults with uncomplicated SSTI in real world clinical practice [15, 16, 18, 33]. Other studies of uncomplicated SSTIs have focused upon antibiotic appropriateness in pediatric patients [17, 20] and in adult patients in ambulatory care settings [19]. Our current evaluation adds to the growing body of evidence demonstrating that the management of hospitalized adult patients with uncomplicated SSTIs represents an opportunity to significantly reduce antimicrobial use by reducing the duration of therapy in addition to promoting the use of narrow spectrum therapy targeting aerobic gram-positive organisms only.

Jenkins and colleagues demonstrated that implementation of a clinical practice guideline for inpatient cellulitis and cutaneous abscess led to shorter durations of more targeted antibiotic therapy without adversely impacting clinical cure rates [33]. They demonstrated a decrease from 13 days to 10 days for median duration of total antibiotic therapy, while showing fewer patients received antimicrobial agents with broad aerobic gram negative activity (66% versus 36%; *P* < 0.001) and broad anaerobic activity (76% versus 49%; *P* < 0.001) [33]. Pasquale et al demonstrated that the addition of formal guidance via prospective audit with feedback by an antimicrobial stewardship program can reduce the use of hospital resources including broad spectrum antibiotic therapy, hospital length of stay, and readmission rates [36]. Their interventions resulted in a reduction in inpatient length of stay from 6.2 days to 4.4 days (*P* < 0.001) while reducing the 30 day readmission rate from 16.7% to 6.5% (*P* = 0.05) [36]. Given the high rates of inappropriate use of antibiotics in terms of both spectrum of therapy and duration of treatment, uncomplicated SSTI management represents an ideal target for enhanced antimicrobial stewardship. Given these findings, our Antimicrobial Stewardship Program (ASP) at each institution implemented a bundled initiative to optimize the management of uncomplicated SSTIs by improving prescribing practices. The bundle included (1) an educational lecture presented to Internal Medicine residency house staff, Internal Medicine medical staff, the department of Hospitalist medicine, and the Division of Infectious Diseases; (2) the creation of an SSTI institutional clinical decision making algorithm which was distributed to medical staff and house staff via our yearly Antimicrobial Guide available in both print and electronic format; and (3) real-time prospective audit of the management of patients with uncomplicated SSTIs by the ASP team with direct oral feedback regarding recommended management changes. Assessment of the impact of this bundled approach is ongoing.

## Conclusions

In summary, uncomplicated SSTIs are a common cause of hospitalizations. Antibiotic therapy is frequently of excessive duration and unnecessarily broad. Optimizing the use of antibiotics for this disease state is a necessity given the ongoing *C. difficile* epidemic and the rise of antimicrobial resistance. An antimicrobial stewardship program offers the potential to enhance the use of resources and outcomes for patients with uncomplicated SSTIs.

## Abbreviations

AGH: Allegheny General Hospital
AHN: Allegheny Health Network
AMA: Against medical advice
ASP: Antimicrobial Stewardship Program
*C. difficile*: *Clostridium difficile*
CD4: Cluster of differentiation 4
CT: Computed tomography
HIV: Human immunodeficiency virus
ICD-9: International Classification of Diseases, Ninth Revision
ICU: Intensive care unit
IDSA: Infectious Diseases Society of America
IV: Intravenous
LOS: Length of stay
MRI: Magnetic resonance imaging
MRSA: Methicillin-resistant *S. aureus*
MSSA: Methicillin-susceptible *S. aureus*
*S. aureus*: *Staphylococcus aureus*
SSTI: Skin and soft tissue infection
WPH: Western Pennsylvania Hospital
